# Calculated plasma volume status is associated with poor outcomes in acute ischemic stroke treated with endovascular treatment

**DOI:** 10.3389/fneur.2023.1229331

**Published:** 2023-07-27

**Authors:** Dixia Zhang, Qiuru Li, Jun Liu, Lijuan Ma, Jing Ye, Guifen Hu, Guangzong Li

**Affiliations:** Department of Neurology, The Sixth People's Hospital of Chengdu, Chengdu, China

**Keywords:** ischemic stroke, endovascular treatment, plasma volume, congestion, prognosis

## Abstract

**Background and purpose:**

The impact of calculated plasma volume status (PVS) on the prognosis of acute ischemic stroke treated with endovascular treatment (EVT) remains undetermined. This study aimed to investigate the association between PVS and 90 days functional outcomes after EVT.

**Methods:**

We enrolled patients treated with EVT in the anterior circulation from a prospective registry. The endpoint was a modified Rankin scale score of ≥3 points at 90 days after EVT. We used multivariable logistic regression models to investigate the association between PVS and poor outcomes. We used the restricted cubic spline to present the linearity between PVS and poor outcomes.

**Results:**

Among the 187 enrolled patients (median age, 65 years; 35.8% women), a total of 81 patients (43.3%) experienced poor outcomes at 90 days. In multivariable analyses, PVS was associated with poor outcomes despite increasing confounding factors (odds ratio, 3.157; 95% confidence interval, 1.942–5.534; *P* < 0.001). The restricted cubic spline revealed a positive correlation between PVS and the risk of poor outcomes after EVT (*P* for nonlinearity = 0.021).

**Conclusion:**

Our study found that an elevated PVS value was associated with poor outcomes after EVT. Further prospective cohorts were warranted to evaluate the utility of PVS in AIS treated with EVT.

## Introduction

Ischemic stroke is the most common cause of death and long-term disability in China (1, 2). Randomized controlled trials have shown that endovascular treatment (EVT) is associated with improved functional independence and can extend the time window for acute ischemic stroke (AIS) patients with large-vessel occlusions in the anterior circulation (3–6). Despite the higher rates of successful recanalization, approximately 50% of AIS patients treated with EVT could not achieve functional independence during the process of rehabilitation (7). Previous studies suggested that the benefits from EVT could be attenuated by the stroke severity, pre-stroke disability, and major complications caused by EVT such as symptomatic intracranial hemorrhage (SICH) (8–10). Hence, it is warranted to have a better understanding of potential predictors to help physicians identify AIS patients with high risks of poor outcomes at the early stage after EVT.

Heart failure is an important risk factor for the short- and long-term prognosis of ischemic stroke. Previous studies have revealed that heart failure was associated with a 2-fold increased risk of mortality (11) and poor functional outcomes at 90 days in patients with ischemic stroke (12). Plasma volume (PV) is a marker of systemic congestion, which is a major complication of heart failure (13). However, accurate measurement of the PV could be challenging in clinical practice due to the technical difficulty and the requirement of invasive tools (14, 15). Notably, plasma volume status (PVS), which is calculated from non-invasive biomarkers of hematocrit and body weight, has been shown to be moderately correlated with gold-standard measurements using radioisotope assays (16). Previous studies have revealed that PVS was associated with clinical outcomes of heart failure (17), transcatheter aortic valve implantation (18), and cardiovascular diseases (19). However, few research studies have investigated the association between PVS and the risk of poor outcomes in AIS patients treated with EVT.

Hence, we performed the current study to investigate the potential association between PVS and poor outcomes in AIS patients treated with EVT.

## Methods

### Study population

This study presented a retrospective analysis based on a prospective registry conducted in the Sixth People's Hospital of Chengdu between August 2018 and September 2022. We included patients under the following criteria: (1) aged ≥18 years; (2) diagnosed as AIS presented with LVO in the anterior circulation (the internal carotid artery or the M1 segment of the middle cerebral artery); (3) received EVT within 6 h of witnessed symptom onset; (4) pre-stroke modified Rankin Scale score (mRS) score of <2. Exclusion criteria were incomplete weight or hematocrit data and follow-up information. This study was approved by the ethics committee of the Sixth People's Hospital of Chengdu and was designed according to the ethical guidelines of the 1964 Declaration of Helsinki and later amendments or comparable ethical standards. Written informed consent was waived due to the retrospective design.

### Data collection

Collected data included demographic characteristics, medical history, laboratory data, transthoracic echocardiography, vital signs, location of occlusion, procedural parameters, stroke severity as assessed by the National Institutes of Health Stroke Scale (20), stroke etiology as assessed by the trial of ORG 10,172 in the acute stroke treatment classification (21), cerebral ischemia as assessed using the Alberta Stroke Program Early CT Score (22), and recanalization status as assessed using the modified thrombolysis in cerebral ischemia score. Heart failure was defined by the Universal Definition and Classification of Heart Failure (23). Brain computed tomography and magnetic resonance images were routinely performed at admission and reperformed within 24 h after the procedure or at any time of neurological deterioration. All radiological images were evaluated by two experienced neurologists who were blinded to the current study. Symptomatic intracranial hemorrhage (SICH) was defined according to the Heidelberg Bleeding Classification (24).

### Definition of PVS

PVS (Hakim formula) was obtained after calculating the actual and ideal PV by using two well-known equations, which have been reported in detail in previous studies (16). Actual PV = {[1– hematocrit] × [a + (b × weight [kg])]}, where hematocrit is a fraction (a = 1,530 and b = 41 in men; a = 864 and b = 47.9 in women). Ideal PV = c × weight (kg), where c=39 in men and c = 40 in women. PVS was subsequently calculated by the deviation of actual PV from ideal PV as follows: PVS = [(actual PV – ideal PV)/ideal PV] × 100%. PVS (Duarte formula) was calculated as follows: ePVS (mL/g) = 100 × (1 – hematocrit)/hemoglobin in g/dL (25).

### Endovascular treatment

EVT was performed by experienced neuro-interventionists using aspiration thrombectomy (Penumbra [Alameda, CA, USA]), stent retrievers [Solitaire (Medtronic, Irvine, CA, USA) or Trevo (Stryker, Fremont, CA, USA)], or the combination of both techniques. Intravenous thrombolysis was performed within 4.5 h after the onset of symptoms. Rescue therapies such as intracranial angioplasty, intra-atrial thrombolysis, stent implementation, or tirofiban administration should be considered when the reperfusion of the target artery fails.

### Endpoint

Functional outcomes were evaluated with the mRS score by trained neurologists who were blinded to this study based on standardized interviews at 90 days. Poor functional outcome was defined as an mRS score of >2 at 90 days.

### Statistical analysis

Normally distributed continuous parameters were presented as mean ± standard deviation (SD) and compared with the *t*-test. Non-normally distributed continuous parameters were presented as median (interquartile range [IQR]) and compared with the Mann–Whitney *U*-test. Categorical parameters were presented as *n* (%) and compared with the chi-square tests or Fisher's exact tests as appropriate. Multiple imputations by chained equations were used to handle missing values.

We applied univariable logistic regression analyses to detect potential predictors for poor outcomes after EVT and used multivariable logistic regression models to verify the association between PVS and poor outcomes. Model 1 was adjusted for age and sex. Model 2 was additionally adjusted for hypertension, diabetes mellitus, atrial fibrillation, hyperlipidemia, coronary heart disease, heart failure, smoking, and drinking. Model 3 was adjusted for variables with a *P*-value of <0.1 in the univariable analysis after the backward stepwise selection method. We treated PVS as a continuous variable and a categorical variable (PVS tertiles) in the logistic regression models, respectively and reported the results as odds ratio (OR) and 95% confidence interval (CI).

We used the receiver operative characteristic curve to evaluate the discriminative performance of PVS for predicting poor outcomes after EVT. To examine the non-linear dose–response relationship between PVS and poor outcome, we used the restricted cubic spline with four knots with adjustment for variables in model 3, and the relationship was non-linear when the *P*-value was <0.05 (26). Net reclassification index (NRI) and integrated discrimination improvement (IDI) can compare the difference in predictive accuracy between the original model and the new model that combines the original model and an additional component. We used NRI and IDI to evaluate the improvement of the discriminative performance after adding PVS into model 3 as a continuous or categorical variable, respectively (27).

All statistical analyses were conducted using R statistical software version 4.2.2. (R Foundation, Vienna, Austria), and a two-sided *P*-value of < 0.05 was considered to be statistically significant.

## Results

### Baseline characteristics of the study population

A total of 187 patients with AIS treated with EVT were included in our study after excluding 26 patients with incomplete clinical data and follow-up information, which consisted of 35.8% female patients, and the median age was 65 years (IQR, 53–72.5). After being divided into three groups according to PVS tertiles, patients with the highest tertiles were more likely to be men, had lower levels of body weight and hematocrit, with higher proportions of smoking, heart failure, number of attempts to achieve successful recanalization, and poor outcomes at 90 days (all *P* < 0.05, Table 1). A total of 81 (43.3%) patients developed poor outcomes after EVT. Patients with poor outcomes were more likely to be older, had lower levels of body weight hematocrit, higher levels of systolic blood pressure, diastolic blood pressure, PVS, fasting blood glucose, stroke severity, higher proportions of atrial fibrillation and heart failure, and lower proportions of good collateral status and successful recanalization (all *P* < 0.05, Supplementary Table 1).

**Table 1:** Baseline characteristics of the study population according to PVS (Hakim formula) tertiles.

**Variables**	**PVS level**			***P* value**
	**T1 < −2.6 (*n =* 62)**	**−2.6 ≤ T2 < 4.6** **(*n =* 58)**	**T3 ≥4.6 (*n =* 67)**	
Age, years	64.0 [55.2, 71.0]	68.0 [55.5, 73.8]	63.0 [52.5, 73.0]	0.316
Female, *n* (%)	33 (53.2)	23 (39.7)	11 (16.4)	< 0.001
Height, cm	170.0 [160.0, 172.0]	166.5 [160.0, 170.0]	165.0 [160.0, 170.0]	0.107
Weight, kg	71.0 [63.0, 75.0]	69.0 [64.0, 75.0]	60.0 [55.0, 66.5]	< 0.001
Systolic blood pressure, mmHg	146.5 [125.8, 161.5]	148.5 [136.5, 160.8]	145.0 [129.5, 158.0]	0.533
Diastolic blood pressure, mmHg	84.5 [74.2, 94.8]	82.0 [75.0, 90.0]	81.0 [75.5, 90.0]	0.732
**Vascular risk factors**, ***n*** **(%)**				
Hypertension	45 (72.6)	37 (63.8)	46 (68.7)	0.585
Diabetes mellitus	15 (24.2)	9 (15.5)	14 (20.9)	0.493
Hyperlipidemia	11 (17.7)	3 (5.2)	6 (9.0)	0.071
Coronary heart disease	13 (21.0)	13 (22.4)	13 (19.4)	0.918
Atrial fibrillation	27 (43.5)	20 (34.5)	19 (28.4)	0.194
Heart failure	0 (0.0)	0 (0.0)	22 (32.8)	< 0.001
Smoking	12 (19.4)	19 (32.8)	27 (40.3)	0.035
Drinking	9 (14.5)	12 (20.7)	17 (25.4)	0.309
**Laboratory data**				
Hemoglobin, g/dL	129.0 [117.2, 140.0]	130.5 [117.8, 143.0]	135.0 [124.5, 144.0]	0.098
Hematocrit (%)	41.3 [38.6, 44.2]	35.3 [33.4, 38.0]	34.1 [32.2, 35.1]	< 0.001
Fasting blood glucose, mmol/L	7.3 [5.5, 8.7]	6.9 [5.7, 8.8]	6.8 [5.8, 8.5]	0.921
Blood urea nitrogen, mmol/L	5.5 [4.5, 6.9]	5.0 [4.1, 7.1]	5.0 [4.1, 6.6]	0.504
Creatinine, μmol/L	73.3 [60.4, 86.9]	70.4 [60.2, 84.7]	74.0 [64.5, 97.0]	0.376
Total cholesterol, mg/dL	4.2 (1.2)	4.5 (1.1)	4.2 (1.0)	0.214
Triglyceride, mg/dL	1.1 [0.7, 1.5]	1.1 [0.7, 1.5]	1.2 [0.9, 1.7]	0.282
High-density lipoprotein, mg/dL	1.1 [1.0, 1.3]	1.2 [1.0, 1.5]	1.1 [0.9, 1.3]	0.501
Low-density lipoprotein, mg/dL	2.6 (0.8)	2.7 (1.0)	2.5 (0.8)	0.471
**TOAST**, ***n*** **(%)**				0.525
Atherosclerosis	30 (48.4)	28 (48.3)	41 (61.2)	
Cardioembolism	27 (43.5)	26 (44.8)	21 (31.3)	
Other etiology	5 (8.1)	4 (6.9)	5 (7.5)	
Prior IVT, *n* (%)	23 (37.1)	17 (29.3)	20 (29.9)	0.585
**Recanalization outcomes**				
Number of attempts, n	1.5 [1.0, 2.0]	2.0 [1.0, 3.0]	2.0 [1.0, 3.0]	0.020
mTICI 2b/3, *n* (%)	51 (82.3)	49 (84.5)	58 (86.6)	0.796
From onset to puncture, min	131.5 [85.0, 182.5]	126.5 [94.0, 194.5]	120.0 [75.5, 146.0]	0.177
From puncture to recanalization, min	75.0 [56.5, 105.8]	88.0 [63.8, 135.0]	81.0 [50.0, 126.0]	0.146
Baseline NIHSS, score	16.0 [11.2, 20.8]	15.5 [12.0, 18.8]	16.0 [10.5, 18.0]	0.742
NIHSS 24h, score	11.0 [6.0, 17.5]	11.5 [7.0, 18.0]	12.0 [6.0, 17.5]	0.843
Baseline ASPECTS, score	9.0 [8.0, 10.0]	9.0 [8.0, 10.0]	9.0 [8.0, 10.0]	0.897
**Procedural parameters**, ***n*** **(%)**				
ASITN/SIR 2-3	34 (54.8)	30 (51.7)	39 (58.2)	0.767
Rescue therapy	30 (48.4)	27 (46.6)	32 (47.8)	0.979
**Occlusion site**				0.602
ICA	24 (38.7)	20 (34.5)	29 (43.3)	
MCA	38 (61.3)	38 (65.5)	38 (56.7)	
SICH, *n* (%)	3 (4.8)	8 (13.8)	9 (13.4)	0.189
mRS at 90 days, score	2.0 [1.0, 2.8]	2.0 [1.0, 5.0]	3.0 [1.0, 4.5]	0.021
mRS >2 at 90 days, *n* (%)	16 (25.8)	26 (44.8)	39 (58.2)	0.001

ASITN/SIR, the American Society of Interventional and Therapeutic Neuroradiology/Society of Interventional Radiology; ASPECTS, the Alberta Stroke Program Early Computed Tomography Score; ICA, internal carotid artery; IVT, intravenous thrombolysis; MCA, middle cerebral artery; mRS, modified Rankin Scale Score; mTICI, modified thrombolysis in cerebral infarction score; NIHSS, National Institute of Health Stroke Scale; PVS, plasma volume status, SICH, symptomatic intracranial hemorrhage; TOAST, the trial of ORG 10172 in acute stroke treatment classification.

### Relationship between PVS and poor outcomes after EVT

In univariable analyses, age (OR, 1.039; 95% CI, 1.014–1.067; *P* = 0.003), systolic blood pressure (OR, 1.021; 95% CI, 1.008–1.035; *P* = 0.002), hypertension (OR, 1.978; 95% CI, 1.046–3.834; *P* = 0.039), atrial fibrillation (OR, 2.027; 95% CI, 1.106–3.747; *P* = 0.023), PVS-Hakim (OR, 1.065; 95% CI, 1.032–1.102; *P* < 0.001), PVS-Duarte (OR, 1.508; 95% CI, 1.095–2.138; *P* = 0.015), heart failure (OR, 3.214; 95% CI, 1.283–8.813; *P* = 0.016), fasting blood glucose (OR, 1.124; 95% CI, 1.037–1.231; *P* = 0.007), TOAST (others vs. atherosclerosis: OR, 0.113; 95% CI, 0.006–0.604; *P* = 0.040), successful recanalization (OR, 0.405; 95% CI, 0.175–0.904 *P* = 0.030), stroke severity (OR, 1.117; 95% CI, 1.064–1.180; *P* < 0.001), cerebral ischemia (OR, 0.712; 95% CI, 0.581–0.854; *P* = 0.001), collateral status (OR, 0.354; 95% CI, 0.193–0.639; *P* = 0.001), and SICH (OR, 9.120; 95% CI, 2.922–40.172; *P* = 0.001) were significantly associated with poor outcomes.

**Figure 1:**
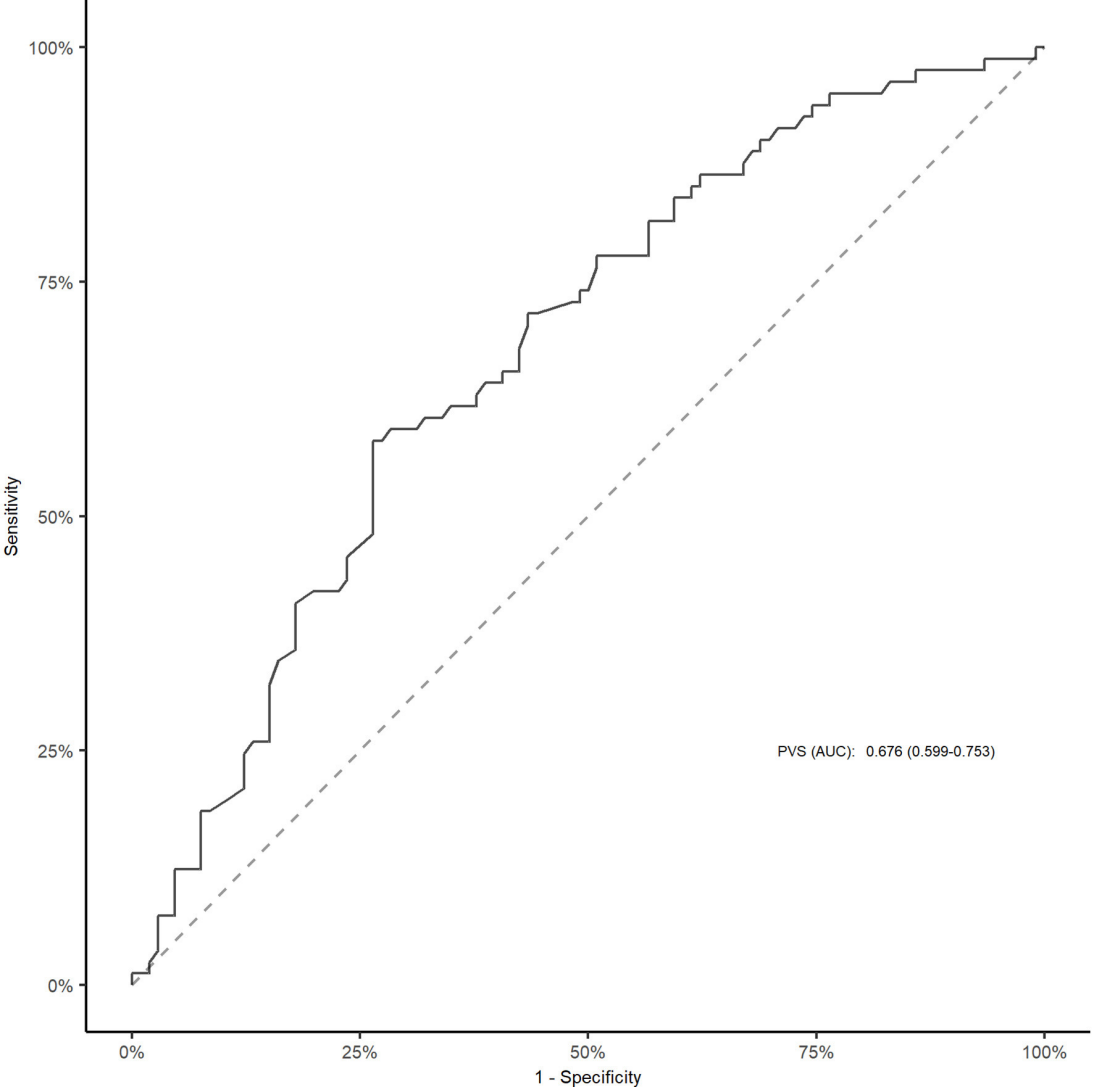
ROC curve for PVS (Hakim formula) to predict poor outcomes after EVT. The cut-off value for PVS was 3.75. EVT, endovascular treatment; ROC, receiver operative characteristic; AUC, area under the curve; PVS, plasma volume status.

**Figure 2:**
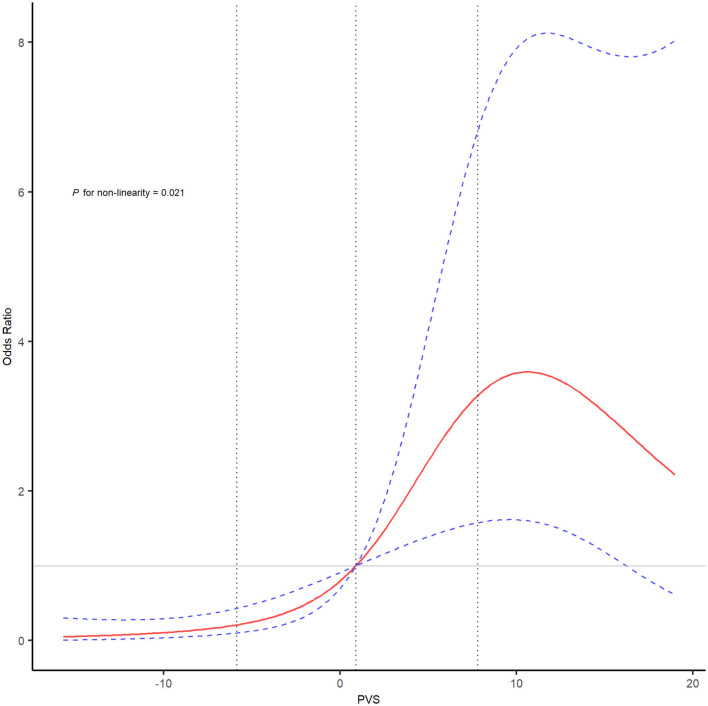
The restricted cubic spline of PVS (Hakim formula) and the risk of poor outcomes after EVT. The restricted cubic spline was adjusted for covariates included in model 3 with four knots (5th, 35th, 65th, and 95th percentiles). The red line showed the odds ratio value, and the blue dotted lines showed the corresponding 95% confidence intervals. The gray dotted lines indicated the first and third percentiles of the PVS value. EVT, endovascular treatment; PVS, plasma volume status.

As shown in Figure 1 and Supplementary Figure 1, the area under the curve was 0.676 (95% CI, 0.599–0.753) for PVS-Hakim and 0.618 (95% CI, 0.537–0.699) for PVS-Duarte to predict poor outcomes after EVT. The optimal cut-off values for PVS-Hakim and PVS-Duarte were 3.75 and 4.99, respectively. The restricted cubic spline revealed that there was a non-linear relationship between PVS-Hakim and poor outcomes (*P* for non-linearity = 0.021, Figure 2) and a linear relationship between PVS-Duarte and poor outcomes (*P* for non-linearity = 0.918, Supplementary Figure 2). In multivariable analyses, PVS-Hakim was significantly associated with poor outcomes in model 1 (OR per SD: 2.227; 95% CI, 1.549–3.306; *P* < 0.001), model 2 (OR per SD: 2.288; 95% CI, 1.567–3.463; *P* < 0.001), and model 3 (OR per SD: 3.157; 95% CI, 1.942–5.534; *P* < 0.001; Table 2 and Supplementary Figure 3), respectively. PVS-Duarte was associated with poor outcomes in model 1 and model 2 (Supplementary Table 3). In sensitivity analysis, 128 patients had undergone transthoracic echocardiography, and the association remained significant after adjustment for left ventricular ejection fractions and inferior vena cava diameters (*P* < 0.05; Supplementary Tables 4, 5). In subgroup analyses, PVS-Hakim was significantly associated with poor outcomes according to stroke subtypes (*P* for interactio*n* = 0.433) and the presence of heart failure (*P* for interactio*n* = 0.070; Supplementary Table 6). Furthermore, PVS- Duarte did not improve the discriminative performance (*P* > 0.05, Supplementary Table 7), while adding PVS-Hakim into model 3 significantly improved the discriminative performance (continuous NRI: 0.411; 95% CI, 0.135– 0.678; *P* < 0.001; categorical NRI: 0.367; 95% CI, 0.137–0.691; *P* = 0.009; IDI: 0.099; 95% CI, 0.056–0.141; *P* < 0.001; Table 3).

**Table 2:** Association between PVS (Hakim formula) and poor outcomes after EVT.

	**PVS (T1)**	**PVS (T2)**	**PVS (T3)**	***P* for trend**	**1-SD increase in PVS**
**Model 1**				< 0.001	
OR (95%CI)	Reference	2.350 (1.062–5.345)	5.108 (2.263–12.158)		2.227 (1.549–3.306)
*P-*value.1		0.037	< 0.001		< 0.001
**Model 2**				< 0.001	
OR (95%CI)	Reference	2.448 (1.057–5.853)	4.84 (1.899–12.983)		2.189 (1.465–3.394)
*P* value.2		0.039	0.001		< 0.001
**Model 3**				< 0.001	
OR (95%CI)	Reference	4.021 (1.339–13.228)	13.259 (4.309–47.491)		3.157 (1.942–5.534)
*P* value.3		0.016	< 0.001		< 0.001

Model 1 was adjusted for age and sex. Model 2 was adjusted for age, sex, hypertension, diabetes mellitus, atrial fibrillation, hyperlipidemia, heart failure, coronary heart disease, smoking, and drinking. Model 3 was adjusted for fasting blood glucose, TOAST, baseline NIHSS, and baseline ASPECTS score. ASPECTS, the Alberta Stroke Program Early Computed Tomography Score; CI, confidence interval; EVT, endovascular treatment; NIHSS, National Institute of Health Stroke Scale; OR, odds ratio; PVS, plasma volume status, TOAST, the trial of ORG 10172 in acute stroke treatment classification.

**Table 3:** Reclassification indexes of PVS (Hakim formula) for poor outcomes after EVT.

**Indexes**	**Estimate (95% CI)**	***P* value**
NRI (continuous)	0.411 (0.135–0.678)	< 0.001
NRI (categorical)	0.367 (0.137–0.691)	0.009
IDI	0.099 (0.056–0.141)	< 0.001

CI, confidence interval; IDI, integrated discrimination improvement; NRI, net reclassification improvement.

## Discussion

In the present study, we found that calculated PVS was significantly associated with poor outcomes in AIS patients treated with EVT, and patients with higher PVS values were more likely to have poor outcomes. Furthermore, by means of reclassification indexes, adding PVS to models improved the discriminative performance and might be useful in clinical prevention and early identification of patients at high risk for potential poor outcomes after EVT.

To date, clinical evidence from meta-analyses supports the fact that EVT is the gold-standard treatment for patients with AIS in the anterior circulation (3). However, the proportion of patients with poor outcomes is still high and might limit the usage of EVT in patients with large infarct core (28). Van de Graaf et al. (7) analyzed 3,180 patients treated with EVT and found that 45% of patients did not achieve functional dependence at 90 days. They proposed that baseline patient characteristics and postprocedural factors were important predictors of poor outcomes (7). Linfante et al. (8) also enrolled 354 patients from a multicenter registry and reported that 49.6% of patients with successful recanalization still had poor outcomes. Duan et al. (29) performed a retrospective analysis in China and revealed that 43.3% of patients had poor outcomes, and the neutrophil–lymphocyte ratio was a significant predictor for clinical outcomes at 90 days.

Calculated PVS is a reliable prognostic predictor for mortality and morbidity in patients with heart failure (17, 25). Previous studies suggested that PVS was associated with complications of heart failure such as systemic congestion and pulmonary edema (30). Higher PVS values were also associated with all-cause and cardiovascular mortality and the addition of PVS into baseline predictors improved the reclassification indexes for mortality (31). Kawai et al. (31) assessed the risk of in-hospital and long-term mortality in acute myocardial infarction according to PVS levels. The results indicated that PVS had an incremental effect on the predictive performance of the GRACE score (19).

In the present study, we found that PVS was associated with poor outcomes after EVT. In multivariable analyses, PVS remained an independent predictor after adjusting for potential covariates including heart failure. Li et al. (12) retrospectively analyzed patients with ischemic stroke with or without heart failure. They found that the left ventricular ejection fraction was significantly associated with 90-day disability and the area under the curve was 0.85 (12). Takahashi et al. (32) further investigated the impact of heart failure on long-term outcomes in AIS patients and conducted a prospective observational study. AIS patients with heart failure had a higher risk of poor outcomes and major adverse cardiovascular events during the long-term follow-up (32). Moreover, systemic congestion could worsen heart failure and is linked to poor prognosis regardless of left ventricular ejection (33). However, the gold-standard measurement of PV requires invasive examinations such as pulmonary artery catheterization or the injection of radioactive molecules. Meanwhile, PVS is a non-invasive biomarker calculated from convenient markers such as hematocrit and body weight. Previous studies suggested that PVS had similar diagnostic values with imaging and hemodynamic congestion markers (34). The findings of our study indicated that PVS could help screen patients at high risk for poor outcomes, and physicians should treat AIS patients with high PVS values with caution.

The mechanisms between PVS and poor outcomes could be due to the following explanations. PV was reported to be closely associated with the renin–angiotensin–aldosterone system, which regulated the renal excretion of sodium and plasma osmolality. Bhalla et al. found that raised plasma osmolality was associated with an increased risk of unfavorable clinical outcomes in AIS patients (35). Moreover, PVS was also related to the presence of erythrocytosis and could increase the risk of thrombo-embolic events (19). Furthermore, the renin–angiotensin–aldosterone system could exacerbate the process of neurohumoral activation and lead to volume overload (13). Abnormal PVS could worsen cardiac function and interfere with blood pressure regulation, which might increase the risk of malignant cerebral edema and lead to poor outcomes (36).

The mechanisms between PVS and poor outcomes in patients with failed recanalization, large artery atherosclerosis, and poor collateral status can be explained in the following way: intravascular volume is an important component in blood pressure and volume overload, and systemic congestion may be associated with progressive edema in ischemic brain tissues, which could lead to increased interstitial pressures and reduced collateral flow to the penumbra and eventually cause collateral failure and hemorrhagic transformation in patients with failed recanalization (37). Volume overload and systemic congestion are also associated with atrial disturbing inflammatory bodies and lead to larger infarct volumes and extra intense hemorrhagic transformation through the activation of atrial traumatic inflammation in patients with poor collateral status (38). Large artery atherosclerosis is the pathological formation of *in situ* thrombosis owing to chronic vascular stenosis and plaque rupture in the culprit artery. An excessive volume load is associated with the release of vasoactive hormones from myocardial tissues, triggering inflammatory responses and hemodynamic instability, which may hinder the initiation of secondary and tertiary circulation and result in an increased stroke severity and hemorrhagic infarction in patients with large artery atherosclerosis (39, 40).

To the best of our knowledge, this was the first study to investigate the association between PVS and clinical outcomes after EVT. First, we performed the retrospective analysis with a small sample size, which might provide residual confounders and a lack of generalizability. Second, we calculated PVS only at admission, while repeated measurements of PVS might be more informative. Third, we could not investigate the potential roles of cardiac variables related to heart failure such as echocardiography parameters and natriuretic peptides, which were not routinely collected in patients treated with EVT. Fourth, we could not compare the gold-standard measurements of PV and the calculated PVS because of costs and the requirements of invasive tools.

In conclusion, our study found that elevated calculated PVS was linked to poor outcomes after EVT. These results suggested that PVS could help physicians screen high-risk patients. Further prospective cohorts are warranted to evaluate the utility of PVS in AIS patients treated with EVT.

## Data availability statement

The raw data supporting the conclusions of this article will be made available by the authors, without undue reservation.

## Ethics statement

This study was approved by the Ethics Committee of the Sixth People's Hospital of Chengdu. Written informed consent for participation was not required for this study in accordance with the national legislation and the institutional requirements.

## Author contributions

DZ, QL, and GL were included in the conceptualization and the design of the study. DZ, JL, LM, and GH acquired and analyzed the data. DZ, JY, and GL were involved in the supervision of the study. DZ, GH, and GL drafted the manuscript. All authors contributed to the article and approved the submitted version.
