# Supplementary material for: Calculated plasma volume status is associated with poor outcomes in acute ischemic stroke treated with endovascular treatment
Source: Front Neurol. 2023 Jul 27;14:1229331. doi: 10.3389/fneur.2023.1229331 (PMC10415678; doi:10.3389/fneur.2023.1229331)
Supplement: Supplementary file 1 [file Data_Sheet_1.PDF]

Supplementary materials

Table 1. Baseline characteristics of the study population.

Variables	Poor outcomes at 90 days			*P* value
	Total (n = 187)	Yes (n = 81)	No (n = 106)	
Age, years	65.0 [53.0, 72.5]	67.0 [59.0, 76.0]	62.0 [53.0, 69.0]	0.004
Female, n (%)	67 (35.8)	30 (37.0)	37 (34.9)	0.883
Height, cm	166 [160, 170]	165 [160, 170]	167 [160, 172]	0.368
Weight, kg	65.5 [60.0, 73.5]	63.5 [59.0, 70.0]	70.0 [61.2, 75.0]	0.001
Systolic blood pressure, mmHg	145.0 [130.0, 160.0]	150.0 [140.0, 160.0]	140.0 [125.0, 158.0]	0.002
Diastolic blood pressure, mmHg	82.0 [75.0, 91.0]	87.0 [77.0, 94.0]	80.0 [74.0, 89.8]	0.027
Vascular risk factors, n (%)				
Hypertension	128 (68.4)	62 (76.5)	66 (62.3)	0.054
Diabetes mellitus	38 (20.3)	18 (22.2)	20 (18.9)	0.703
Hyperlipidemia	20 (10.7)	5 (6.2)	15 (14.2)	0.131
Coronary heart disease	39 (20.9)	21 (25.9)	18 (17.0)	0.190
Atrial fibrillation	66 (35.3)	36 (44.4)	30 (28.3)	0.033
Heart failure	22 (11.8)	15 (18.5)	7 (6.6)	0.023
Smoking	58 (31.0)	28 (34.6)	30 (28.3)	0.448
Drinking	38 (20.3)	15 (18.5)	23 (21.7)	0.725
Laboratory data				
Hemoglobin, g/dL	132.0 [121.5, 143.0]	132.0 [123.0, 142.0]	132.0 [120.0, 143.0]	0.813
Hematocrit (%)	36.0 [34.0, 39.4]	35.1 [33.3, 38.0]	37.7 [34.4, 41.2]	0.001
PVS-Hakim (%)	0.9 (10.0)	4.2 (9.0)	-1.5 (10.0)	<0.001
PVS-Duarte (%)	4.9 (0.9)	5.1 (1.0)	4.8 (0.9)	0.012
Fasting blood glucose, mmol/L	7.0 [5.6, 8.7]	7.4 [6.1, 10.3]	6.6 [5.3, 8.2]	0.003
Blood urea nitrogen, mmol/L	5.2 [4.2, 6.9]	5.1 [4.4, 7.3]	5.3 [4.1, 6.6]	0.623
Creatinine, µmol/L	73.0 [62.9, 90.6]	72.4 [63.0, 99.0]	73.4 [62.2, 85.8]	0.424
Total cholesterol, mg/dL	4.3 (1.1)	4.4 (1.0)	4.2 (1.2)	0.269
Triglyceride, mg/dL	1.1 [0.7, 1.6]	1.1 [0.7, 1.5]	1.2 [0.8, 1.6]	0.298

High-density lipoprotein, mg/dL	1.1 [1.0, 1.3]	1.2 [1.0, 1.4]	1.1 [1.0, 1.3]	0.268
Low-density lipoprotein, mg/dL	2.6 (0.9)	2.7 (0.9)	2.6 (0.9)	0.423
TOAST, n (%)				0.004
Atherosclerosis	99 (52.9)	40 (49.4)	59 (55.7)	
Cardioembolism	74 (39.6)	40 (49.4)	34 (32.1)	
Other etiology	14 (7.5)	1 (1.2)	13 (12.3)	
Prior IVT, n (%)	60 (32.1)	22 (27.2)	38 (35.8)	0.270
Recanalization outcomes				
Number of attempts, n	2.0 [1.0, 3.0]	2.0 [1.0, 3.0]	2.0 [1.0, 3.0]	0.125
mTICI 2b/3, n (%)	158 (84.5)	63 (77.8)	95 (89.6)	0.044
From onset to puncture, min	125.0 [85.0, 178.0]	125.0 [83.0, 167.0]	127.5 [88.5, 183.8]	0.549
From puncture to recanalization, min	80.0 [55.0, 125.0]	80.0 [50.0, 141.0]	80.5 [60.0, 111.5]	0.963
Baseline NIHSS, score	16.0 [12.0, 19.0]	17.0 [14.0, 21.0]	13.5 [10.0, 17.8]	<0.001
NIHSS 24h, score	11.0 [6.0, 18.0]	18.0 [12.0, 30.0]	8.0 [3.0, 11.8]	<0.001
Baseline ASPECTS, score	9.0 [8.0, 10.0]	9.0 [7.0, 10.0]	10.0 [8.2, 10.0]	0.001
Procedural parameters, n (%)				
ASITN/SIR 2-3	103 (55.1)	33 (40.7)	70 (66.0)	0.001
Rescue therapy	89 (47.6)	44 (54.3)	45 (42.5)	0.144
Occlusion site				0.140
ICA	73 (39.0)	37 (45.7)	36 (34.0)	
MCA	114 (61.0)	44 (54.3)	70 (66.0)	
SICH, n (%)	20 (10.7)	17 (21.0)	3 (2.8)	<0.001

Abbreviations: ASITN/SIR, the American Society of Interventional and Therapeutic Neuroradiology/Society of Interventional Radiology; ASPECTS, the Alberta Stroke Program Early Computed Tomography Score; ICA, internal carotid artery; IVT, intravenous thrombolysis; MCA, middle cerebral artery; mRS, modified Rankin Scale Score; mTICI, modified Thrombolysis in Cerebral Infarction Score; NIHSS, National Institute of Health Stroke Scale; PVS, plasma volume status, SICH, symptomatic intracranial hemorrhage; TOAST, the trial of ORG 10172 in Acute Stroke Treatment classification.

Table 2. Univariate logistic regression for the risk of poor outcomes after EVT.

Variables	OR (95% CI)	*P* value
Age	1.039 (1.014-1.067)	0.003
Gender	0.912 (0.499-1.670)	0.763
Height	0.981 (0.943-1.020)	0.333
Weight	0.965 (0.936-0.994)	0.022
Systolic blood pressure	1.021 (1.008-1.035)	0.002
Diastolic blood pressure	1.018 (0.998-1.039)	0.090
Vascular risk factors		
Hypertension	1.978 (1.046-3.834)	0.039
Diabetes mellitus	1.229 (0.597-2.515)	0.573
Hyperlipidemia	0.399 (0.125-1.083)	0.089
Coronary heart disease	1.711 (0.842-3.510)	0.138
Atrial fibrillation	2.027 (1.106-3.747)	0.023
Heart failure	3.214 (1.283-8.813)	0.016
Smoking	1.338 (0.717-2.500)	0.359
Drinking	0.820 (0.390-1.683)	0.593
Laboratory data		
Hemoglobin	1.001 (0.986-1.016)	0.923
Hematocrit	0.890 (0.824-0.956)	0.002
PVS-Hakim	1.065 (1.032-1.102)	<0.001
PVS-Duarte	1.508 (1.095-2.138)	0.015
Fasting blood glucose	1.124 (1.037-1.231)	0.007
Blood urea nitrogen	0.990 (0.916-1.044)	0.718
Creatinine	1.007 (0.999-1.017)	0.117
Total cholesterol	1.162 (0.893-1.521)	0.268
Triglyceride	0.907 (0.677-1.183)	0.486
High-density lipoprotein	1.007 (0.557-1.773)	0.980
Low-density lipoprotein	1.144 (0.824-1.594)	0.421
TOAST		
Atherosclerosis	Reference	
Cardioembolism	1.735 (0.947-3.205)	0.076
Other etiology	0.113 (0.006-0.604)	0.040
Prior IVT	0.667 (0.352-1.246)	0.208
Recanalization outcomes		
Number of attempts	1.102 (0.907-1.346)	0.329
mTICI 2b/3	0.405 (0.175-0.904)	0.030
From onset to puncture,	0.998 (0.994-1.002)	0.394
From puncture to recanalization	1.001 (0.996-1.007)	0.581
Baseline NIHSS	1.117 (1.064-1.180)	<0.001
NIHSS 24h	1.200 (1.138-1.278)	<0.001
Baseline ASPECTS	0.712 (0.581-0.854)	0.001
Procedural parameters		
ASITN/SIR 2-3	0.354 (0.193-0.639)	0.001

Rescue therapy	1.612 (0.902-2.899)	0.108
Occlusion site		
ICA	Reference	
MCA	0.612 (0.336-1.106)	0.105
SICH	9.120 (2.922-40.172)	0.001

Abbreviations: ASITN/SIR, the American Society of Interventional and Therapeutic Neuroradiology/Society of Interventional Radiology; ASPECTS, the Alberta Stroke Program Early Computed Tomography Score; CI, confidence interval; EVT, endovascular treatment; ICA, internal carotid artery; IVT, intravenous thrombolysis; MCA, middle cerebral artery; mRS, modified Rankin Scale Score; mTICI, modified Thrombolysis in Cerebral Infarction Score; NIHSS, National Institute of Health Stroke Scale; OR, odds ratio; PVS, plasma volume status, SICH, symptomatic intracranial hemorrhage; TOAST, the trial of ORG 10172 in Acute Stroke Treatment classification.

Table 3. Association between PVS (Duarte formula) and poor outcomes after EVT.

	PVS (T1)	PVS (T2)	PVS (T3)	*P* for trend	1-SD increase in PVS
Model 1				0.041	
OR (95%CI)	Reference	1.654 (0.764-3.622)	2.888 (1.321-6.485)		1.421 (1.025-2.016)
P value.1		0.203	0.009		0.041
Model 2				0.032	
OR (95%CI)	Reference	1.643 (0.725-3.785)	2.657 (1.157-6.266)		1.464 (1.043-2.103)
P value.2		0.237	0.023		0.032
Model 3					
OR (95%CI)	Reference	0.939 (0.344-2.532)	1.831 (0.679-4.989)	0.220	1.275 (0.872-1.921)
P value.3		0.901	0.232		0.220

Model 1 was adjusted for age and sex.

Model 2 was adjusted for age, sex, hypertension, diabetes mellitus, atrial fibrillation, hyperlipidemia, heart failure, coronary heart disease, smoking, and drinking.

Model 3 was adjusted for fasting blood glucose, TOAST, baseline NIHSS and baseline ASPECTS score.

Abbreviations: ASPECTS, the Alberta Stroke Program Early Computed Tomography Score; CI, confidence interval; EVT, endovascular treatment; NIHSS, National Institute of Health Stroke Scale; OR, odds ratio; PVS, plasma volume status, TOAST, the trial of ORG 10172 in Acute Stroke Treatment classification.

Table 4. Association between PVS (Hakim formula) and poor outcomes after EVT in patients who had undergone TTE.

	PVS (T1)	PVS (T2)	PVS (T3)	*P* for trend	1-SD increase in PVS
Model 1				0.002	
OR (95%CI)	Reference	2.201 (0.798-6.331)	4.626 (1.649-13.934)		2.142 (1.367-3.536)
P value.1		0.132	0.005		0.002
Model 2				0.002	
OR (95%CI)	Reference	2.315 (0.746-7.515)	5.383 (1.65-19.114)		2.286 (1.385-4.008)
P value.2		0.151	0.007		0.002
Model 3					
OR (95%CI)	Reference	5.236 (0.877-40.608)	23.486 (3.929-218.69)	0.001	4.057 (1.887-10.534)
P value.3		0.084	0.002		0.001

Model 1 was adjusted for age, sex, LVEF, and IVC diameters.

Model 2 was adjusted for age, sex, hypertension, diabetes mellitus, atrial fibrillation, hyperlipidemia, heart failure, coronary heart disease, smoking, drinking, LVEF, and IVC diameters.

Model 3 was adjusted for fasting blood glucose, TOAST, baseline NIHSS, baseline ASPECTS score, LVEF, and IVC diameters.

Abbreviations: ASPECTS, the Alberta Stroke Program Early Computed Tomography Score; CI, confidence interval; EVT, endovascular treatment; IVC, inferior vena cava; LVEF, left ventricular ejection fractions; NIHSS, National Institute of Health Stroke Scale; OR, odds ratio; PVS, plasma volume status, TOAST, the trial of ORG 10172 in Acute Stroke Treatment classification; TTE, trans-thoracic echocardiography.

Table 5. Association between PVS (Duarte formula) and poor outcomes after EVT in patients who had undergone TTE.

	PVS (T1)	PVS (T2)	PVS (T3)	*P* for trend	1-SD increase in PVS
Model 1				0.009	
OR (95%CI)	Reference	2.513 (0.912-7.242)	4.168 (1.516-12.239)		1.929 (1.21-3.258)
P value.1		0.079	0.007		0.009
Model 2				0.014	
OR (95%CI)	Reference	2.747 (0.903-8.942)	4.438 (1.503-14.165)		1.937 (1.179-3.384)
P value.2		0.081	0.009		0.014
Model 3					
OR (95%CI)	Reference	0.660 (0.126-3.213)	2.704 (0.597-13.358)	0.115	1.826 (0.942-4.166)
P value.3		0.609	0.203		0.115

Model 1 was adjusted for age, sex, LVEF, and IVC diameters.

Model 2 was adjusted for age, sex, hypertension, diabetes mellitus, atrial fibrillation, hyperlipidemia, heart failure, coronary heart disease, smoking, drinking, LVEF, and IVC diameters.

Model 3 was adjusted for fasting blood glucose, TOAST, baseline NIHSS, baseline ASPECTS score, LVEF, and IVC diameters.

Abbreviations: ASPECTS, the Alberta Stroke Program Early Computed Tomography Score; CI, confidence interval; EVT, endovascular treatment; IVC, inferior vena cava; LVEF, left ventricular ejection fractions; NIHSS, National Institute of Health Stroke Scale; OR, odds ratio; PVS, plasma volume status, TOAST, the trial of ORG 10172 in Acute Stroke Treatment classification; TTE, trans-thoracic echocardiography.

Table 6. Subgroup analysis according to TOAST and heart failure.

	OR (95% CI)	*P* value	*P* for interaction
TOAST			0.433
Atherosclerosis	1.062 (1.016-1.115)	0.011	
Cardioembolism	1.093 (1.037-1.164)	0.002	
Heart failure			0.070
Yes	1.701 (1.149-3.335)	0.045	
No	1.051 (1.017-1.088)	0.004	

Abbreviations: CI, confidence interval; OR, odds ratio; TOAST, the trial of ORG 10172 in Acute Stroke Treatment classification.

Table 7. Reclassification Indexes of PVS (Duarte formula) for poor outcomes after EVT.

Indexes	Estimate (95% CI)	*P* value
NRI (continuous)	0.015 (-0.023- 0.141)	0.736
NRI (categorical)	-0.020 (-0.060-0.258)	0.809
IDI	0.007 (-0.007-0.021)	0.320

Abbreviations: CI, confidence interval; IDI, integrated discrimination improvement; NRI, net reclassification improvement.

Figure 1. ROC curve for PVS (Duarte formula) to predict poor outcomes after EVT.

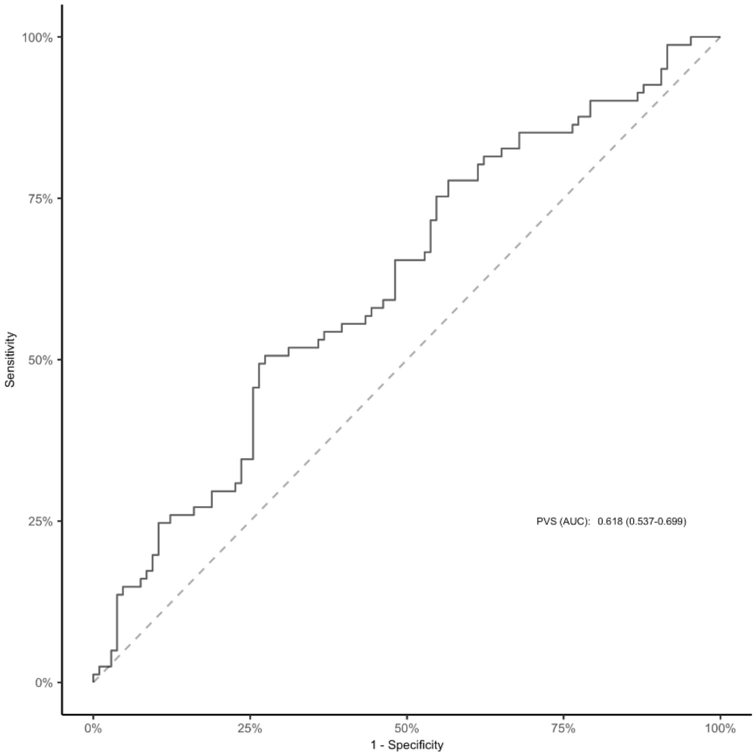

Abbreviations: The cut-off value for PVS was 4.99. EVT, endovascular treatment; ROC, receiver operative characteristic; AUC, area under the curve; PVS, plasma volume status.

Figure 2. The restricted cubic spline of PVS (Duarte formula) and the risk of poor outcomes after EVT.

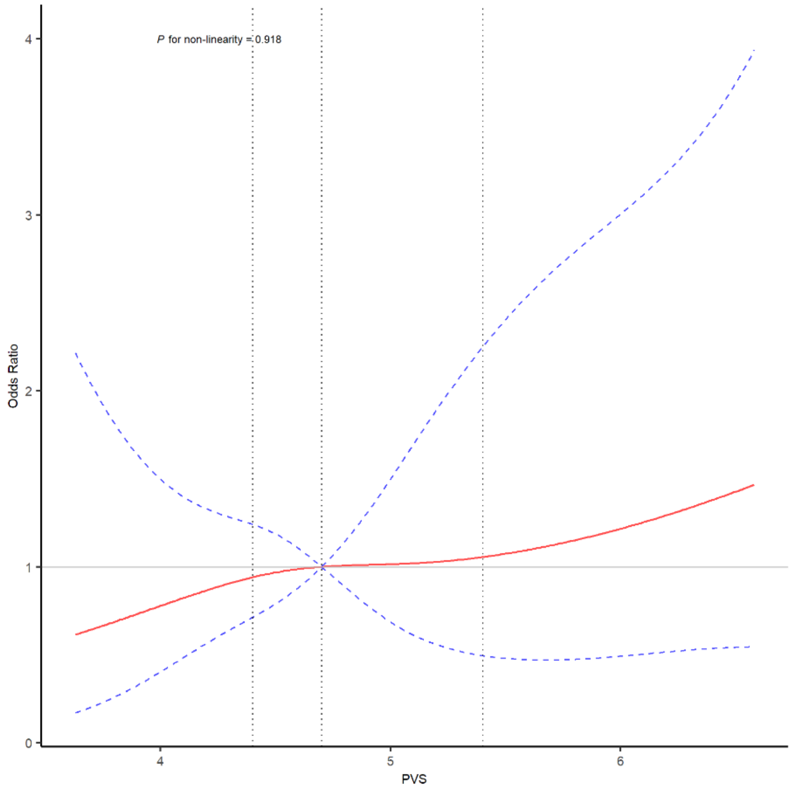

Abbreviations: EVT, endovascular treatment; PVS, plasma volume status.

Figure 3. Odds ratio of other predictors for poor outcomes in model 3.

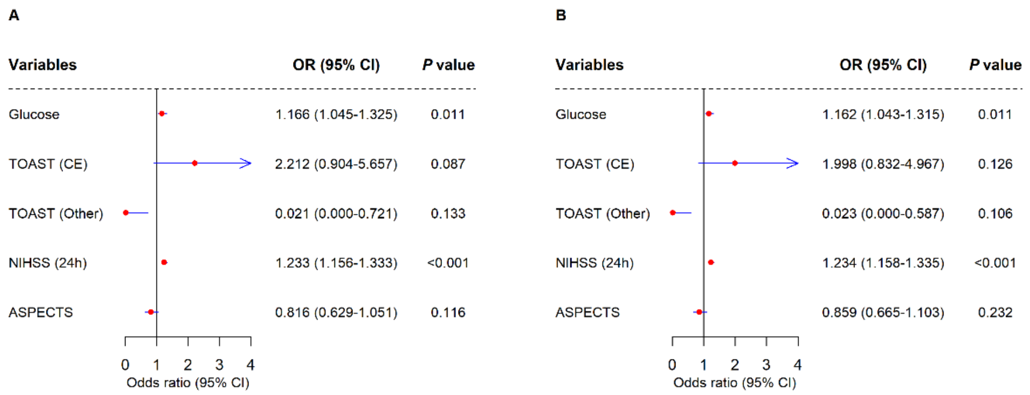

Abbreviations: PVS was regarded as a categorical variable in A and as a continuous variable in B. ASPECTS, the Alberta Stroke Program Early Computed Tomography Score; NIHSS, National Institute of Health Stroke Scale; TOAST, the trial of ORG 10172 in Acute Stroke Treatment classification.
